# Bibliography

**Published:** 1894-10

**Authors:** 


### Bibliography.

A Manual of Dental Anatomy, Human and Comparative. By Charles S. Tomes, M.A., F.R.S., with two hundred and thirty-five Illustrations. Fourth Edition. P. Blakiston, Son & Co., Philadelphia, 1894.

The student of comparative anatomy has now the way made very easy for him as compared with that of the one struggling with the subject thirty years ago. Then expensive works or tedious search in libraries, in addition to extended personal investigation, were required to meet the demands of special training.

This was, perhaps, not a matter for regret, as it forced the dental student to a direct examination of tooth-forms, and in this way accomplishing a more thorough comprehension of dental anatomy than could be obtained from books. Yet these cannot be dispensed with, and the student of to-day should feel grateful to the author and publisher of this volume that it is made possible for him, at this period, to add to his library at moderate cost the substance of Owen's great work and the researches in comparative dental anatomy brought up to date.

In the preface to this fourth edition the author very modestly says, "It has been my endeavor to give an outline of the more important facts of odontology, such as may serve as an introduction to a more extended study of the subject." This hardly does justice to this work. It covers all essentials so thoroughly that the dental student has no cause to look further, as far as books are concerned. It, however, remains true, that books, no matter how ably prepared, should not take the place of personal and direct investigation.

Chapters I. to VIII. include the consideration of The Nature of Teeth, Dental Tissues, Development of Teeth, Attachment, etc. The author has been very successful in explaining all the processes of tooth-formation, and that with admirable clearness. He has avoided the temptation of most authors of overloading with technical terms, and where these are used they are explained in a way to relieve the subject of all difficulty.

On examination of the pages devoted to the histology of structure, one is impressed with the fact that for years very little, perhaps nothing, has been added to our knowledge of the hard tissues of the teeth. There has been much work in this direction, but the new points evolved, while possibly true, still require confirmation. We are no further advanced in the study of the sensitiveness of dentine and the function of the fibril than we were when John Tomes, the discoverer, explained their presence in the tubuli of dentine. It is true we know more of the contents of these tubes, and are familiar with the appearance of "Neumann's sheaths," and have a clearer idea of the inner tubular contents, but exactly how sensation is conveyed is not explained, for, as the author says, "no true nerve-fibril has yet been proved to enter the dentine."

The chapter on "Development of Teeth" is made very plain. It is a difficult subject to clearly describe on paper, but the author, by following development from the teeth of fishes to those of mammalia, renders the subject easy of comprehension to the student. He has enriched his pages by the most recent researches, while not omitting those which, as he says, "might be regarded as classical."

Beginning with Chapter IX., page 219, the author describes the typical forms of the fishes, reptiles, birds, and mammals. There is, therefore, compressed in this "Manual of Dental Anatomy" all the essentials to the study of this branch of comparative anatomy, bringing this important subject within the reach of every one at a moderate cost.

As it is equally necessary for the practitioner to have a book of ready reference, as well as the student one for study, this volume, the work of Charles S. Tomes, can be recommended, without any reservations, as the most complete of its kind now available, and should be placed upon the list of text-books of all colleges and in dental libraries.

A Treatise on Pyorrhœa Alveolaris: A System for its Prompt, Positive, and Permanent Cure. By Junius E. Cravens, D.D.S. W. M. Herriott, Publisher, Indianapolis, Ind.

This monograph, of forty-eight pages, gives the author's ideas of pyorrhœa alveolaris, but does not add materially to our comprehension of this pathological condition. This does not seem to have been his intention in writing the book, but rather to give his mode of treatment.

His general proposition, "that wherever a tooth retains good attachment upon one or more sides of its root it may safely be classed as curable," possibly cannot be controverted; but it does not necessarily follow that those suffering from greater destruction cannot be restored to health.

The origin of calculus, according to the author's belief, may be ascribed to the constant bathing of pus, and he is doubtless correct, as the proportion of lime-salts in pus greatly exceeds that in saliva.

There is no reasonable cause given for the destruction of the pericementum, beyond the deposition of calculus, for, he says, "As a result of continued periostitis the wall of the socket is gradually destroyed. . . . Just how the pericementum may be affected . . . is not definitely known."

Pus-pockets are at times connected with "galleries" and "grottoes;" the latter are excavations between the roots of molars.

The treatment consists of, first, repeated douches of hot water, heated at 140° F. The part is next subjected to an anæsthetic, cocaine preferred. The root is then scraped with a scaler, to remove all deposits. The case is then treated with a ten-per-cent. solution of nitrate of silver, followed by an astringent, chloride of aluminum, or, preferably, *bromo-chloralum.*

No allusion to systemic conditions is made by the author, and it is presumed he regards pyorrhœa alveolaris as of purely local origin.

A Modern Wizard. By Rodrigues Ottolengui, author of "Artist in Crime," etc. G. D. Putnam's Sons, New York, 1894. For sale by J. B. Lippincott Company, Philadelphia.

Novels, ordinarily, are not the character of books suitable for review in a professional journal, but the above title represents not only the work of a prominent dentist, but contains within it matter very suggestive to the thinker along legal, medical, and psychical lines.

The book opens with the trial of a certain Dr. Medjora, charged with poisoning his wife with morphine. The complication was that she was affected at the same time with diphtheria. The case as presented in its legal aspects is, to the layman's mind, skilfully worked out, ending in the acquittal of the doctor.

The point which the author evidently wishes to be impressed on the reader is the possibility of microbic cultures of contagious diseases being used to destroy life, and thus evade the keenest search of the chemical expert.

The plot is also made to bring into play various psychic powers, some recognized and others still to be proved. Hypnotism plays a prominent part, but the author carries this beyond what is believed to be its legitimate possibilities, and thus creates what must be regarded as a wrong impression. Hypnosis has its limit. Its production in the most susceptible person is usually slow, and the deeper manifestations are not produced at once upon an untrained subject.

The book is written in the author's attractive style, and maintains the interest from the beginning to the end. It is questionable whether he succeeds in drawing his characters very distinctively. Character-building, such as Dickens pre-eminently possessed, can be attained by few. The author makes the boy Leon, fresh from the country and but poorly educated, talk as learnedly as the profound doctor, and the young Agnes follows in the same strain. This is doubtless essential to a proper discussion of certain problems of philosophical thought, but it detracts in a measure from the artistic finish of the production.

The book belongs to the class of which Balzac was the earlier and Marie Corelli the latter representative. They all carry psychic problems to an extreme. In this case the developments in bacteriology are extended beyond the expectations of the most sanguine, and in a very unpleasant way, and yet the possibilities, it would seem, are not over stated.

The book can be read with interest by professional men generally.

